# Genome Sequencing of up to 6,000-Year-Old *Citrullus* Seeds Reveals Use of a Bitter-Fleshed Species Prior to Watermelon Domestication

**DOI:** 10.1093/molbev/msac168

**Published:** 2022-07-30

**Authors:** Oscar A Pérez-Escobar, Sergio Tusso, Natalia A S Przelomska, Shan Wu, Philippa Ryan, Mark Nesbitt, Martina V Silber, Michaela Preick, Zhangjun Fei, Michael Hofreiter, Guillaume Chomicki, Susanne S Renner

**Affiliations:** Royal Botanic Gardens, Kew, Richmond TW9 3AE, United Kingdom; Faculty of Biology, Division of Genetics, University of Munich (LMU), 82152 Planegg-Martinsried, Germany; Royal Botanic Gardens, Kew, Richmond TW9 3AE, United Kingdom; Boyce Thompson Institute, Ithaca, NY 14853, USA; Royal Botanic Gardens, Kew, Richmond TW9 3AE, United Kingdom; Royal Botanic Gardens, Kew, Richmond TW9 3AE, United Kingdom; Faculty of Biology, Systematic Botany and Mycology, University of Munich (LMU), 80638 Munich, Germany; Faculty of Mathematics and Natural Sciences, Institute for Biochemistry and Biology, University of Potsdam, 14476 Potsdam, Germany; Boyce Thompson Institute, Ithaca, NY 14853, USA; USDA-ARS, Robert W. Holley Center for Agriculture and Health, Ithaca, NY 14853, USA; Faculty of Mathematics and Natural Sciences, Institute for Biochemistry and Biology, University of Potsdam, 14476 Potsdam, Germany; Ecology and Evolutionary Biology, School of Bioscience, University of Sheffield, Western Bank, Sheffield S10 2TN, United Kingdom; Faculty of Biology, Systematic Botany and Mycology, University of Munich (LMU), 80638 Munich, Germany; Department of Biology, Washington University, Saint Louis, MO 63130, USA

**Keywords:** watermelon, domestication, population genomics, ancient DNA, C-14-dated African seeds, Neolithic settlements in Libya

## Abstract

Iconographic evidence from Egypt suggests that watermelon pulp was consumed there as a dessert by 4,360 BP. Earlier archaeobotanical evidence comes from seeds from Neolithic settlements in Libya, but whether these were watermelons with sweet pulp or other forms is unknown. We generated genome sequences from 6,000- and 3,300-year-old seeds from Libya and Sudan, and from worldwide herbarium collections made between 1824 and 2019, and analyzed these data together with resequenced genomes from important germplasm collections for a total of 131 accessions. Phylogenomic and population-genomic analyses reveal that (1) much of the nuclear genome of both ancient seeds is traceable to West African seed-use “egusi-type” watermelon (*Citrullus mucosospermus*) rather than domesticated pulp-use watermelon (*Citrullus lanatus* ssp. *vulgaris*); (2) the 6,000-year-old watermelon likely had bitter pulp and greenish-white flesh as today found in *C. mucosospermus*, given alleles in the bitterness regulators *ClBT* and in the red color marker *LYCB*; and (3) both ancient genomes showed admixture from *C. mucosospermus*, *C. lanatus* ssp. *cordophanus*, *C. lanatus* ssp. *vulgaris*, and even South African *Citrullus amarus*, and evident introgression between the Libyan seed (UMB-6) and populations of *C. lanatus*. An unexpected new insight is that *Citrullus* appears to have initially been collected or cultivated for its seeds, not its flesh, consistent with seed damage patterns induced by human teeth in the oldest Libyan material.

## Introduction

The emergence of agriculture has been associated with hotspots of plant domestication, referred to as domestication centers (Vavilov 1926). Although domestication centers in the Fertile Crescent in the Middle East, the Ganges basin, the Yangtze and Yellow River basins, and Central and South America have been accepted since the 1930s, regions of plant domestication in subtropical and tropical Africa, aside from Ethiopia, were not discussed seriously until the 1970s (Harlan 1971). Over the past 50 years, archaeobotanical excavations and carbon-14 (C-14) dates have revealed Neolithic cultures in parts of the Sahara, when the rainfall was greater than now and the flora was largely Mediterranean in nature (Cremaschi and Di Lernia 1999; Richter et al. 2017; Pausata et al. 2020). Thus, sorghum (*Sorghum bicolor*), one of the best-studied crops in Saharan Africa (Wasylikowa and Dahlberg 1999; Mercuri et al. 2018; Smith et al. 2019), was domesticated in the Eastern Sahel (Winchell et al. 2017, 2018), pearl millet (*Cenchrus americanus*) in a region corresponding to northern Mali and Mauritania (Burgarella et al. 2018), and African rice (*Oryza glaberrima*) and yam (*Dioscorea rotundata*) also in West Africa (Manning et al. 2011; Cubry et al. 2018; Scarcelli et al. 2019).

Here we investigate the early history of one of the oldest African crops, the watermelon (*Citrullus lanatus*). Despite the completion of a first reference genome in 2013 (Guo et al. 2013), resequencing of numerous cultivars and landraces (Guo et al. 2019; Wu et al. 2019), and the identification of a likely wild progenitor from Sudan (Renner et al. 2021), the early (defined as prior to 1900) genetic contributions of wild watermelons to the modern sweet-pulped ssp. *vulgaris* remain unclear, mostly because of insufficient geographic sampling. Geographic hypotheses of watermelon domestication have focused on three regions: South Africa, West Africa, and Northeast Africa. The idea of a South African origin predominated between 1930 and 2013, and was derived from Bailey’s (1930) synonymization of Linnaeus’s name for the watermelon, which Linnaeus knew only from specimens prepared from plants cultivated in Italy, with the South African citron melon, collected and named by one of Linnaeus’s students. DNA sequences from the Linnean herbarium in Uppsala as well as representative samples from throughout the genus range, however, revealed that no South African watermelons are closely related to the sweet-pulp watermelon (Chomicki and Renner 2015). Instead, *Citrullus mucosospermus*, also called egusi-type melons, with bitter pulp and fat-rich seeds appeared closer to the sweet-pulp watermelon (Guo et al. 2013; Chomicki and Renner 2015). In Sub-Saharan Africa, the oleaginous seeds of certain watermelons (genus *Citrullus*), melons (*Cucumis*), and white-seed melons (*Cucumeropsis mannii*) are used to thicken “egusi” soups as well as being dry-roasted and eaten as snacks (Schaefer and Renner 2010; Achigan-Dako et al. 2015).

**Fig. 1.**
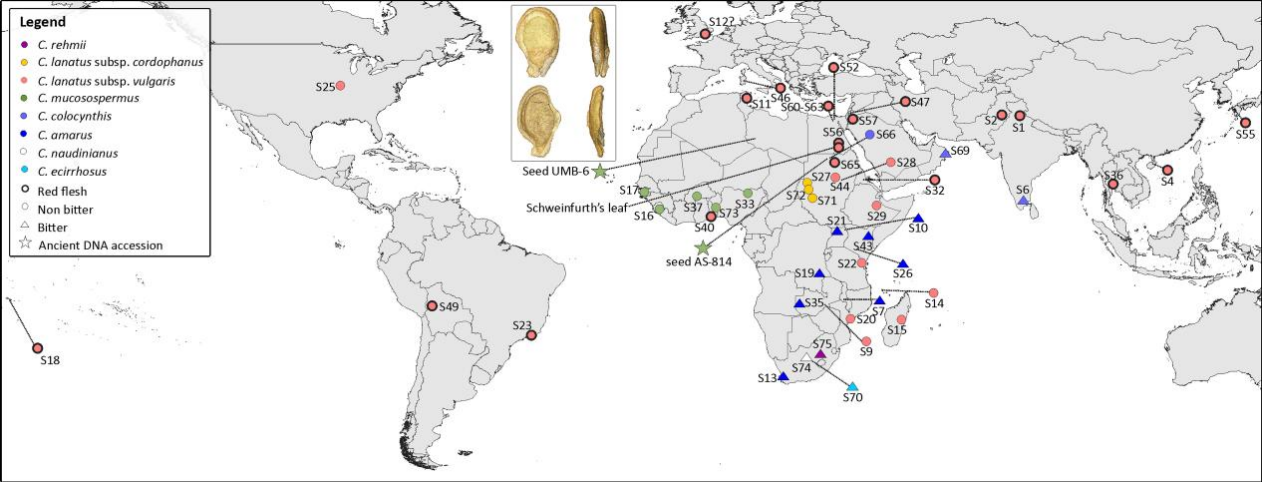
Geographic origin of the ancient DNA samples (stars) and historical *Citrullus* accessions used in this study. We also show the geography of two key fruit traits: flesh color (red flesh: bold border; non-red flesh: thin border) and fruit bitterness (bitter: triangle; non-bitter: circle), based on diagnostic SNPs in the bitterness regulator *ClBT* and Lycopene cyclase (*LYCB*) genes (see Results and Discussion). The inset shows computed-tomography scans of two UMB-6 seeds from Uan Muhuggiag, Libya, radiocarbon dated to 6,182–6,001 calibrated years before present (from Wolcott et al. 2021), which based on morphology had been identified as *Citrullus lanatus.* The lower seed shows a breakage patterns characteristic of modern watermelon seeds cracked by human teeth.

Other data instead point to watermelon deriving from a Northeast African progenitor, namely the sweet-pulped, small-fruited (about 23 × 21 cm diam.), white-fleshed Kordofan melon (*C. lanatus* ssp. *cordophanus*), grown by local farmers in the Kordofan and Darfur regions of Sudan (Ter-Avanesyn 1966; Fursa and Gavrilyuk 1990; Paris 2015; Renner et al. 2017, 2021; Chomicki et al. 2020). Northeast Africa is supported by phylogenomic analyses that included numerous domesticated watermelons, Sudanese *cordophanus* samples, and wild species (Renner et al. 2021). Limestone reliefs in Egyptian tombs that show large, entire fruits displayed on trays next to sweet fruits, such as grapes, suggest that raw watermelons were consumed by the Middle Kingdom, 4,000–3,600 BC (Renner et al. 2021). However, *Citrullus* seeds, mixed with dried *Ziziphus spina-christi* fruits, which are sweet, have been found in numerous baskets in the tomb of Tutankhamun (Hepper 2009), indicating that the seeds were also eaten as a snack. The oldest *Citrullus* seeds so far reported come from a Neolithic settlement in Libya (Wasylikowa and van der Veen 2004), and some show traces of cracking from human teeth (Cox and van der Veen 2008; Wolcott et al. 2021; our fig. 1, inset).

During the African Humid Period (14,800–5,500 years ago; Pausata et al. 2020), which overlaps with the Neolithic and Earliest Bronze Age, the ranges of North African watermelon taxa probably were larger than they are today. This could have led to hybridization, given that all species in the genus *Citrullus*—endemic in Africa, with one extending to the Middle East and India—have the same haploid chromosome number of *n* = 11 (Renner et al. 2017). Modern attempts to enhance disease resistance have involved successful crossing of South African citron melons (*Citrullus amarus*) with sweet-fleshed ssp. *vulgaris* (Orton 1911; Parris 1949; McGregor 2012; McCreight et al. 2013), further suggesting that gene flow may have played a role during the early domestication stages.

In this study, we provide a phylogenomic and population-genomics framework including (1) 6,000- and 3,300-year-old C-14-dated seeds attributed to *C. lanatus* from archeological sites in Libya and northern Sudan; (2) 47 newly sequenced genomes from geographically widespread herbarium specimens collected between 1824 and 2019; and (3) 73 resequenced modern accessions of *Citrullus* from Chinese and American germplasm collections and 9 from greenhouse-cultivated representatives of all species of *Citrullus* (Guo et al. 2019; Renner et al. 2021). Specifically, we asked the following questions: (1) Based on their DNA, can the ancient seeds be assigned to taxa recognized today? (2) Do archaeogenomic data help resolve the early-stage domestication history of the sweet-fleshed watermelon (*C. lanatus* ssp. *vulgaris*)? (3) Given that there is good understanding of specific genes determining fruit bitterness and color (Shang et al. 2014; Zhou et al. 2016; Zhang et al. 2020), can pulp phenotypes be inferred for any of the ancient genomes? (4) Is there evidence for gene flow among different *Citrullus* forms/species, and if so, can introgression be traced in the ancient genomes?

## Results and Discussion

### DNA Sequencing of Two Ancient Seed Genomes

The oldest remains ever assigned to *Citrullus* are seeds from the Libyan Uan Muhuggiag Neolithic settlement (labeled UMB-6 in fig. 1). Based on morphology, these uncharred, desiccated seeds have been identified as *C. lanatus* (Wasylikowa and van der Veen 2004). The Tadrart Acacus mountain range was occupied by pastoralists during the early- to mid-Holocene (van der Veen 1995), and the layer in which the seeds were found was radiocarbon-dated to 7,784–7,545 BP and 8,162–7,520 calBP (calibrated years before the present), whereas one of the Uan Muhuggiag seeds was radiocarbon dated to 5,313 ± 23 BP or 6,182–6,001 calBP (Wolcott et al. 2021), confirming burial during the late Stone Age (supplementary fig. S1, Supplementary Material online). Other (desiccated) watermelon seeds are from a desert encampment (2-R-65) near the pharaonic town of Amara West in northern Sudan (AS-814 in fig. 1); these were radiocarbon-dated in this study to 3,197 ± 23 BP or 3,455–3,373 cal BP (supplementary fig. S1, Supplementary Material online).

When we radiocarbon-dated a supposedly ancient watermelon leaf from Kew’s Economic Botany collection (specimen 40730; Materials and Methods), however, it turned out to date from the 1880s (supplementary fig. S1, Supplementary Material online). Its collector, G. Schweinfurth (1836–1925), worked in one of the tombs at Deir el-Bahari (modern Bahri) just North of Luxor in 1883 from where he sent botanical material to Kew in February 1883. Since Schweinfurth (1884) describes how he moistened and spread out a large watermelon leaf found in one of the tombs to compare it with modern *Citrullus* leaves, it is plausible that one of the latter leaves got mixed up with ancient samples.

We obtained ∼234, 135, and 80 million reads from the Libyan and Sudanese seeds (UMB-6, AS-814) and the Schweinfurth leaf, respectively (supplementary table S1, Supplementary Material online). Nucleotide mis-incorporations indicative of DNA damage predominantly occurred toward both ends of the reads in the DNA read fragments from the Libyan and Sudanese seeds (supplementary fig. S2, Supplementary Material online). Read length distributions were centered on 25–29 bp, consistent with sequencing data from similarly aged plant material (Mascher et al. 2016: ca. 6,000-year-old barley grains; Ramos-Madrigal et al. 2016: a 5,310-year-old corn cob; Vallebueno-Estrada et al. 2016: a 5,300-year-old corn cob). Following this authentication, we estimated the endogenous ancient DNA (aDNA) content by computing the proportion of reads matching the nuclear and plastids reference genomes of *C. lanatus* ssp. *vulgaris.* About 75% of reads were deemed endogenous for the Schweinfurth leaf (from 1883), compared with 22% and ∼1% for the Libyan and Sudanese seeds, respectively. Given age-related damage patterns observed in the Libyan and Sudanese aDNA reads, we quantified the effect of mis-incorporations by computing a transition/transversion ratio (Ti/Tv) and compared the error rate with that in modern damage-free DNA samples. Both analyses were conducted with a base recalibration following the post-mortem DNA damage estimated by mapDamage (see Materials and Methods). The Ti/Tv ratio of the Sudanese seed (AS-814) based on sites genotyped in this sample was 0.873, slightly above the range of modern samples genotyped at the same set of sites (0.806–0.824, SD = 0.010). The result for the Libyan seed (UMB-6) was 1.858, which is identical to the computed mean for modern samples genotyped at sites common with those in the Libyan seed (1.858, SD = 0.0075). This suggests that C > T and G > A substitutions are overall not elevated in the ancient samples (supplementary fig. S2, Supplementary Material online). In addition, a comparison of the excess of derived alleles (i.e., error rates) among modern individuals of *C. mucosospermus* and the Libyan UMB-6 seed genome revealed that the proportion of derived alleles in UMB-6 (1.2%, supplementary fig. S2, Supplementary Material online) is comparable with that of modern individuals (0.86–1.06%), confirming limited mis-incorporations in the ancient sample, unlikely to bias any downstream analysis.

### Phylogenetic Placement of the Ancient Seed Genomes

To infer the taxonomic relatedness of the ancient seeds (UMB-6 and AS-814), we analyzed their partial nuclear and near-complete plastid genome assemblies together with 47 newly sequenced herbarium collections made between 1824 and 2019, and with previously resequenced samples from diverse germplasm and greenhouse-grown representatives of all species of *Citrullus* (Guo et al. 2019; Renner et al. 2021). The taxon names, georeferenced locations, and year of collecting of all 123 newly or previously sequenced herbarium-preserved plants, cultivated plants in germplasm collections, ancient seed samples from Libya and Sudan, and the (supposed) ancient leaf from Kew’s Economic Botany collection (specimen 40730) are shown in supplementary table S2, Supplementary Material online. For the 47 historical herbarium specimens, we sequenced an average of 239 million reads, leading to an average coverage of 56× (supplementary table S1, Supplementary Material online). Figure 1 visualizes the geographic origin of the material used in this study.

**Fig. 2.**
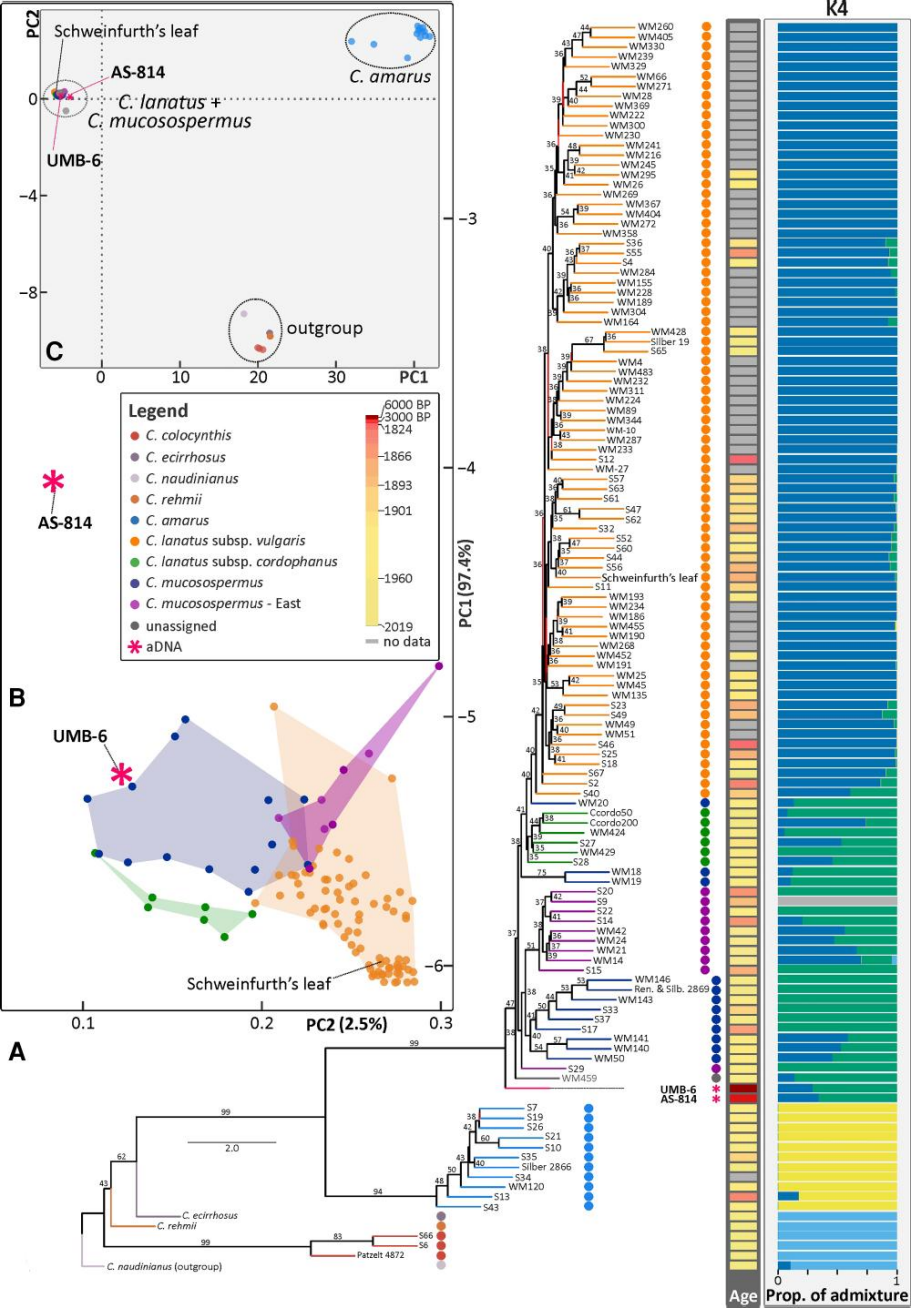
Phylogeny and principal component analyses (PCAs) of 123 *Citrullus* samples. (*A*) Multispecies coalescence phylogeny for 123 accessions of *Citrullus* including 47 historical herbarium-derived genomes (collected between 1824 and 2019), ancient Libyan and Sudanese seeds (UMB-6, AS-814), and Schweinfurth’s leaf from the vicinity of Luxor dating from 1883 (see supplementary table S2, Supplementary Material online, for vouchers). The phylogeny was inferred from 1,431 non-overlapping nuclear 10 Kb blocks derived from 11 chromosomes (*Methods*). Numbers above branches represent posterior probability. The first column to the right of the phylogeny shows the age of the samples, the second admixture at *K* = 4 (see supplementary fig. S5, Supplementary Material online, for *K* = 2–8). (*B*) PCAs based on estimated nuclear genotype likelihoods of all samples. (*C*) A zoom in of the PCA displayed on *A*, focusing on a cluster containing *C. lanatus* ssp. *vulgaris*, *C. lanatus* ssp. *cordophanus*, *C. mucosospermus*, and the aDNA Libyan and Sudanese samples.

The phylogenetic relationships recovered from the plastid genomes were in line with those revealed in previous studies (supplementary fig. S3, Supplementary Material online; Chomicki and Renner 2015; Renner et al. 2021). The partial nuclear genomes of the ancient seed samples (average coverage of 0.04× for AS-814 and 3.7× for UMB-6; supplementary table S1, Supplementary Material online) permitted the inclusion of only UMB-6 in a nuclear phylogeny of *Citrullus*. A multispecies coalescence (MSC) tree from 1,431 maximum likelihood (ML) trees derived from non-overlapping 10 Kb blocks, representing 14.3 Mb of the *Citrullus* nuclear genome placed the UMB-6 seed as sister to a clade of *C. mucosospermus*, *C. lanatus* ssp. *cordophanus*, and *C. lanatus* ssp. *vulgaris* (fig. 2, local posterior probability: 99%).

**Fig. 3.**
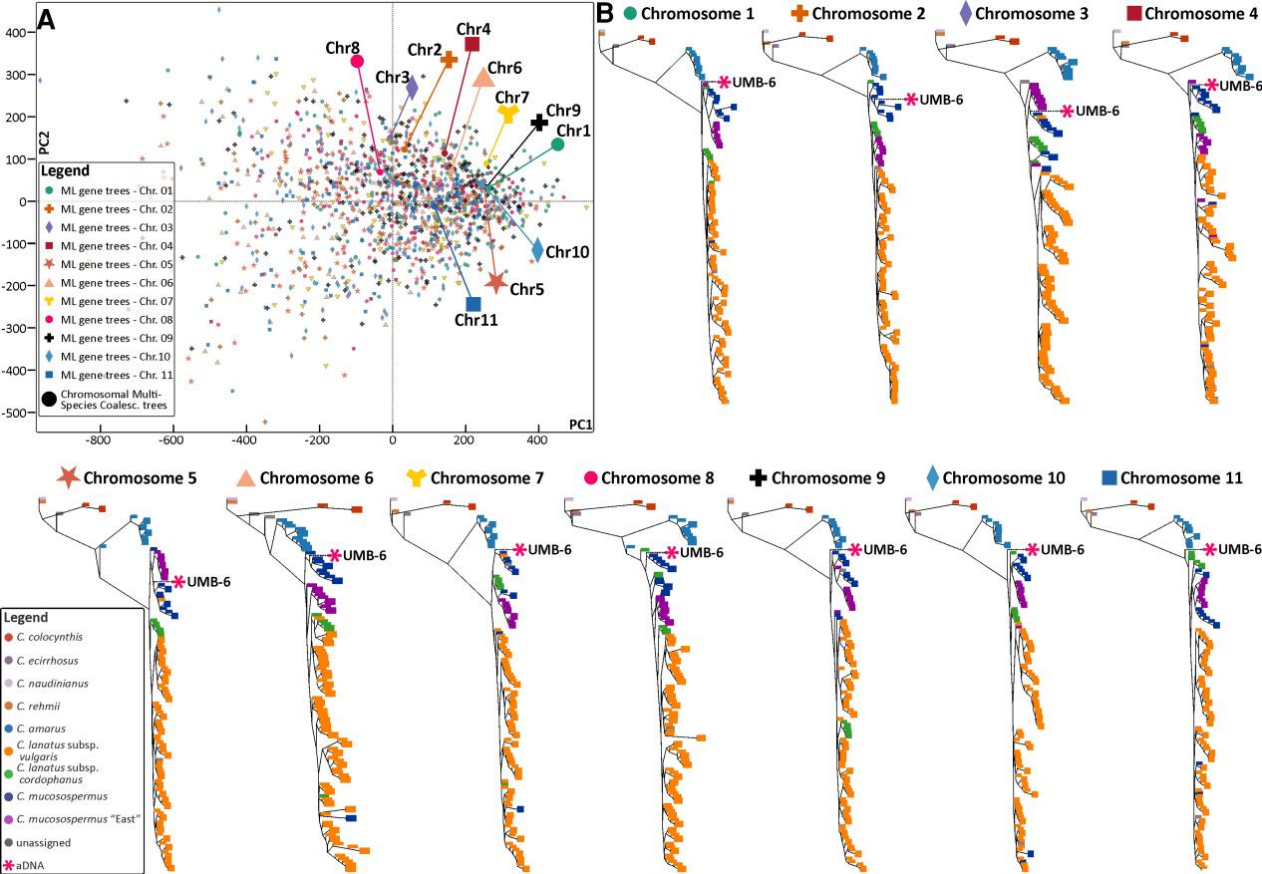
Relationships of the 6,000-year-old Libyan seed with historical and modern accessions of *Citrullus* as inferred by phylogenetic analyses and gene-tree and species-tree space analysis. (A) Maximum likelihood (ML) and Multispecies coalescent (MSC) species-tree space. Each minute dot represents an individual ML tree derived from non-overlapping nuclear 10 Kb alignments, with its color and shape referring to which chromosome block it is derived from. Larger symbols represent MSC trees inferred in ASTRAL from chromosomal ML gene trees (B) ASTRAL MSC phylogenies per chromosome, showing the placement of the ancient Libyan seed UMB-6. The larger symbols above trees represent the corresponding ASTRAL MSC phylogenies inferred for the chromosomal ML gene trees.

Subsequently, we generated phylogenies from each of the 11 watermelon chromosomes (fig. 3). Chromosomes 1, 7, 8, 9, 10, and 11 grouped the ancient Libyan genome (UMB-6) as a sister to *C. lanatus* (with its ssp. *vulgaris* and *cordophanus*) and *C. mucosospermus*, whereas chromosomes 2, 3, 4, 5, and 6 grouped UMB-6 with accessions of *C. mucosospermus.* The normalized quartet score values obtained from the individual trees from each chromosome scaffold indicated that the proportion of gene tree incongruence across chromosomes was similar (average *Q* = 0.658, *n* = 11 [0.64–0.68]).

Most of the phylogenies inferred from the 11 chromosome scaffolds (fig. 3) show a clade of East African accessions of *C. mucosospermus* from Mozambique, Tanzania, the Comoro Islands, Ethiopia, and Madagascar (herbarium samples S9, S14, S15, S20, and S22 and germplasm samples WM14, WM21, WM24, and WM42). These samples also formed a clade in the phylogeny from the non-overlapping 10 Kb blocks (fig. 2). This Eastern *C. mucosospermus* clade has never been recovered before and lacks a formal taxonomic name. Only two East African samples, S29 and WM459, fell outside the clade.

Within the sweet-pulp watermelon clade (ssp. *vulgaris*), the first-diverging sample (fig. 2) was a plant collected in Togo, West Africa, in 1901, followed by a clade grouping most herbarium samples collected between 1892 and 1927 in Northeast Africa (Egypt, Sudan, Eritrea), except for one from Tunisia, as well as other samples from Bolivia (S49, cultivated), Brazil (S23, probably cultivated), the USA (S25, cultivated), Pakistan (S2, cultivated), Samoa (S18, feral, growing on a beach), Sardinia (S46, status unclear), and Israel (S47, feral). *Citrullus lanatus* landraces, the seeds of which are maintained by local farmers rather than being bought from seed companies, in both phylogenies (figs. 2 and 3) group basal to the Chinese (17) and American (12) germplasm collections, countries that have the largest breeding programs, as well as many samples from other countries (Japan, 3 accessions; Mexico, 3; Russia, 3; Hungary, 2; Ukraine, 2; Bulgaria, 1; Egypt, 1; Greece, 1; India, 1; Italy, 1; Philippines, 1; Portugal, 1; Spain, 1; Romania, 1; Turkey, 1; Uzbekistan, 1).

### Ancestry and Introgression History of Modern and Ancient *Citrullus*

To trace the relationships of the Libyan and Sudanese ancient seeds (UMB-6 and AS-814), and test for gene flow among different forms of *Citrullus*, we conducted model-free (i.e., PCA) analyses, model-based ancestry estimation, and explicit topological tests of species-tree incongruence (ABBA-BABA). We also compared the tree topologies derived from the biparentally inherited nuclear genome and the maternally inherited plastid genome. These analyses relied on the calculation of nuclear and plastid genome-wide genotype likelihoods (GLs) derived from the same accessions included in the nuclear and plastid phylogenomic analyses, while accounting for missing data, read coverage, and base and mapping quality as implemented in ANGSD v.098 (see Materials and Methods).

The PCA of the nuclear and plastid genomic data revealed a clear differentiation between individuals of *C. amarus*, the other wild species (*Citrullus colocynthis*, *Citrullus ecirrhosus*, *Citrullus naudinianus*, *Citrullus rehmii*), and the *C. lanatus* clade (*C. mucosospermus*, C. *lanatus* ssp. *cordophanus*, *C. lanatus* ssp. *vulgaris*). Regardless of the proportion of missing data in the analyses, the clustering patterns of the ancient seeds (UMB-6 and AS-814) remained stable (fig. 2*A*, supplementary fig. S4, Supplementary Material online).

Our model-based ancestry analysis showed that at *K* = 4 (the best-fitting *K*, supplementary fig. S5, Supplementary Material online shows *K* = 2–8), introgression was most evident in accessions of *C. lanatus* ssp. *cordophanus*, and West and East African *C. mucosospermus* (e.g., WM140, WM141). Both the 6,000-year-old Libyan seed (UMB-6) and the 3,300-year-old Sudanese seed (AS-814) showed admixture from *C. mucosospermus*, *C. mucosospermus* East, and *C. lanatus* ssp. *vulgaris*.

**Fig. 4.**
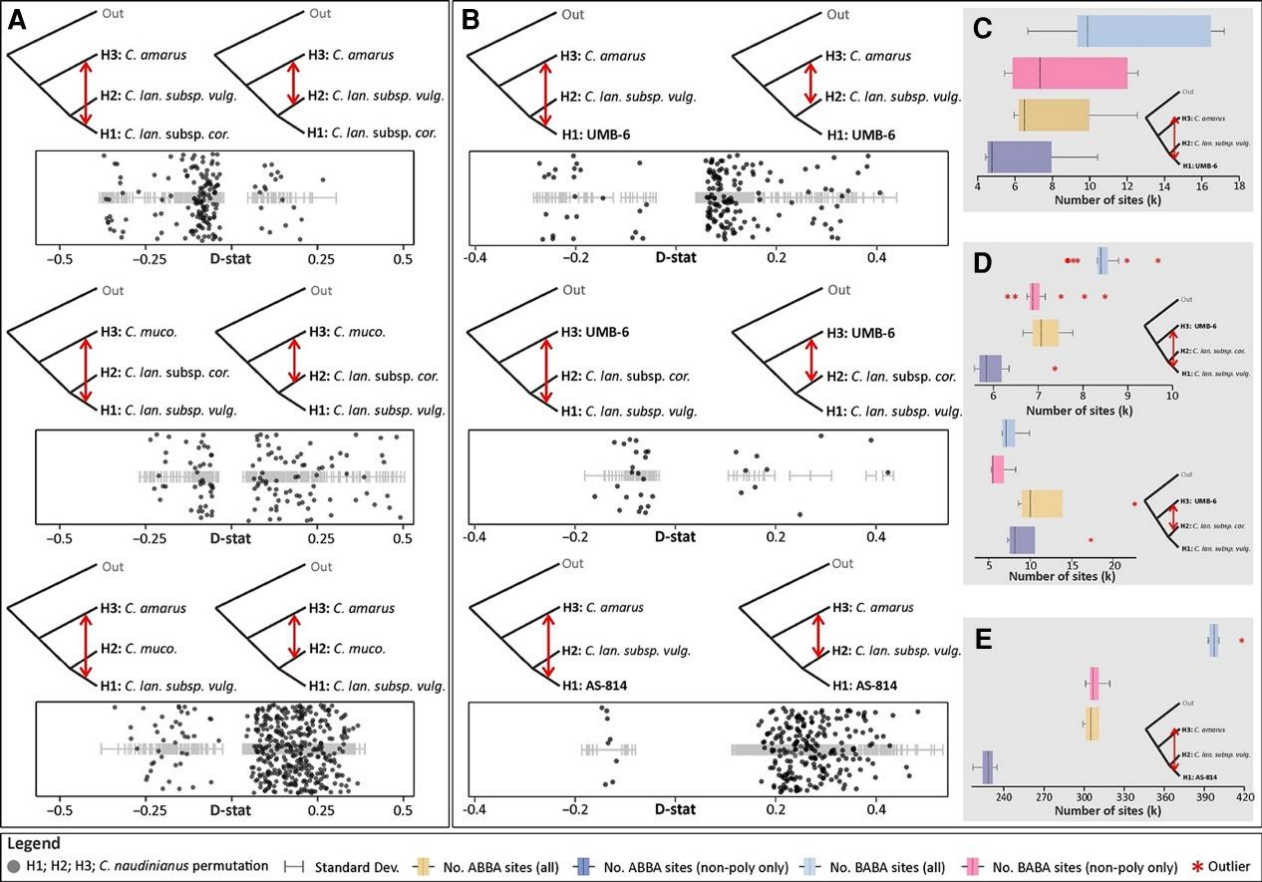
ABBA-BABA test of introgression among the watermelon and its close relatives including the ancient DNA samples. (*A*) Tests of introgression among *C. lanatus* ssp. *vulgaris*, *C. lanatus* ssp. *cordophanus*, *C. amarus*, and *C. mucosospermus* based on all informative sites. (*B*) Test of introgression among the 6,000-year-old Libyan seed (UMB-6), the 3,000-year-old Sudanese seed (AS-814), and *C. lanatus* ssp. *vulgaris*, *C. lanatus* ssp. *cordophanus*, and *C. amarus* based on all informative sites. (*C*) Number of ABBA-BABA sites indicating gene flow between the Libyan seed and *C. amarus*. (*D*) Number of ABBA-BABA sites indicating gene flow between the Libyan seed (UMB-6), *C. lanatus* ssp. *vulgaris*, and *C. lanatus* ssp. *cordophanus*. (*E*) Number of ABBA-BABA sites indicating gene flow between the Sudanese seed and *C. amarus*. The plots presented in *C*, *D*, and *E* are inferred from all sites or non-polymorphic sites only (*D*-values, *Z*-scores, and sample IDs used in all ABBA-BABA tests are provided on supplementary table S3, Supplementary Material online).

For the ABBA-BABA tests, we used all 8–11 million nucleotide sites with an average of ∼6,000 sites per permutation or only non-polymorphic sites, accounting for outgroup and deamination biases (Barlow et al. 2020; see Materials and Methods; supplementary table S3, Supplementary Material online). We ran tests with or without the aDNA, with the groups considered being *C. amarus, C. lanatus* ssp. *vulgaris*, *C. lanatus* ssp. *cordophanus*, *C. mucosospermus*, and the ancient Libyan and Sudanese seed genomes (fig. 4, supplementary table S3, Supplementary Material online). The analyses using all sites and those using only non-polymorphic sites revealed congruent gene flow patterns (supplementary table S3, Supplementary Material online), indicating that outgroup choice and aDNA damage were not biasing the gene flow tests. Tests including only modern individuals revealed introgression between individuals of the East African Kordofan melon (*C. lanatus* ssp. *cordophanus*), the domesticated *C. lanatus* ssp. *vulgaris*, and the egusi-type melon, *C. mucosospermus*, as well as between *C. mucosospermus* and the South African bitter melon, *C. amarus* (fig. 4*A*, supplementary table S3, Supplementary Material online). Tests including the ancient seeds, revealed introgression between the Libyan plant (UMB-6) and *C. amarus*, *C. lanatus* ssp. *vulgaris*, *C. lanatus* ssp. *cordophanus* (fig. 4*B*).

### Comparative Genomics of Pulp-Bitterness and Flesh-Color Genes Reveals that the 6,000-Year-Old Libyan Watermelon was Used for its Seeds, Not its Pulp

**Fig. 5.**
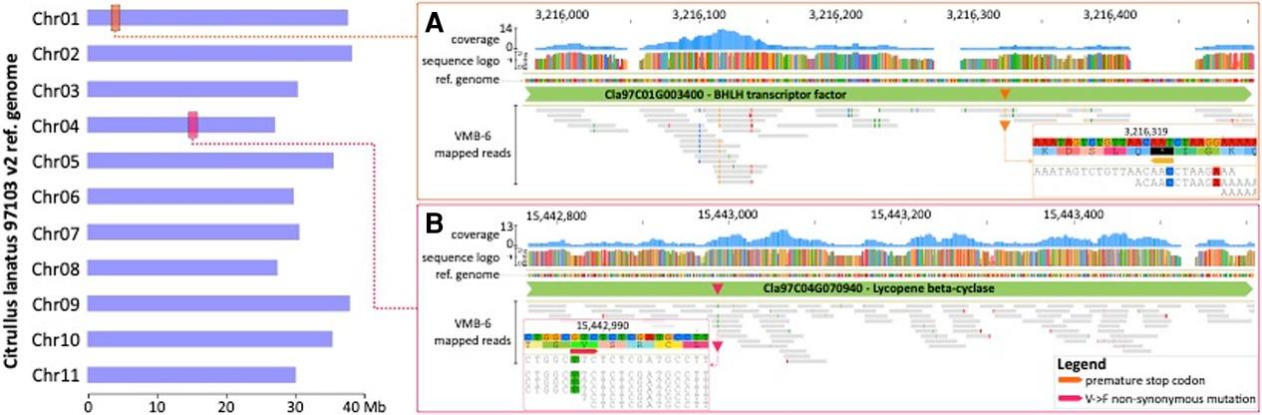
The fruits of the 6,000-year-old Libyan seeds were bitter and greenish-white fleshed. (*A*) Position and coverage of the bitterness regulator *ClBT* gene in UMB-6. (*B*) Position and coverage of the lycopene cyclase gene (*LYCB*) in UMB-6. See also supplementary fig. S6, Supplementary Material online.

Analysis of the bitterness regulator gene *ClBT* in the genomes of *C. amarus*, *C. colocynthis, C. ecirrhosus*, *C. mucosospermus*, *C. rehmii*, and *C. naudinianus* showed that they all contain the bitter allele (fig. 5, supplementary fig. S6, Supplementary Material online), and we also found the bitter allele in the 6,000-year-old Libyan seed (fig. 5). By contrast, the 47 *C. lanatus* plants collected between 1824 and 2019, as well as all four *C. lanatus* ssp. *cordophanus* accessions, had the non-bitter allele. An unusual mutation was discovered in a *C. colocynthis* plant (S66) collected in 2015 from Ernetta Island, in the Abri region of North Sudan (near the site that yielded seed AS-814): Colocynths usually have extremely bitter pulp, but this particular plant (or population) independently had lost pulp bitterness (supplementary fig. S6, Supplementary Material online).

The presence or absence of a mutation (V226F) in the lycopene cyclase (*LYCB*) gene is a proxy for red pulp in watermelons (Bang et al. 2007; Grassi et al. 2013; Zhang et al. 2020). As for *ClBt*, we could identify *LYCB* in the Libyan seed (UMB-6), but not in the Sudanese seed (AS-814), which yielded much less endogenous DNA. We found that the Libyan seed lacks the V226F mutation, implying that the plant’s pulp was not red fleshed (fig. 5). All *C. mucosospermus* accessions lacked the red-flesh marker (supplementary fig. S6, Supplementary Material online). Interestingly, one accession from Saint Louis, USA, collected in 1882 and identified as *C. lanatus* ssp. *vulgaris* (voucher S25) also lacked the red-flesh marker (supplementary fig. S6, Supplementary Material online).

## Conclusion

This study reveals the Neolithic use in Libya of a form of *Citrullus* that was genetically close to today’s seed use, bitter-fleshed, egusi-type watermelon (*Citrullus mucosospermus*), now restricted to Ghana, Benin, and Nigeria in West Africa (Achigan-Dako et al. 2015). The likely use of the Libyan seeds as a snack matches the traces of cracking from human teeth found in a computer-tomographic study of seeds from the same site (Cox and van der Veen 2008; Wolcott et al. 2021; fig. 1, inset). This use of *Citrullus* seeds as a snack appears to have occurred prior to, and independently of, the domestication of pulp-use watermelons from non-bitter progenitor populations (*C. lanatus* ssp. *cordophanus*), presumably in the mid- to late-Holocene middle Nile valley in Sudan, based on the funeral imagery from elite tombs in Middle and New Kingdom Egypt. Our phylogenomic analyses as well as the analyses of the bitterness regulator *ClBt* and lycopene cyclase gene *LYCB* imply that the pulp of the 6,000-year-old Libyan watermelon was whitish and bitter, matching the inference that this plant was used for its nutritious seeds, instead of its pulp. It is unclear, however, whether these plants were cultivated or instead grew wild around the Uan Muhuggiag settlement. Use of a wild, not yet domesticated, plant would fit with other findings about the Neolithic people living at Uan Muhuggiag who were herding Barbary sheep (*Ammotragus lervia*) and cultivating *Panicum laetum* and *Echinochloa colona*, but apparently never fully domesticated any animals or plants (Mercuri et al. 2018).

The East African *C. mucosospermus* clade newly revealed in this study contains interesting examples of wild forms of watermelons used for food in historic times, prior to modern watermelon breeding, which began in 1900 (Orton 1911; Parris 1949). These include herbarium sample S20 from Mozambique, collected in 1860 and described on its label as “cultivated,” and S15 from Madagascar collected in 1882, described as “a globular melon with white stripes running pole to pole, cultivated.” Our work thus unveils the use of *Citrullus* forms from the Neolithic into the premodern era. Future work could involve a pangenome approach, which would allow dissecting the genetic uniqueness of forms that have been used over the past 6,000 years, which in turn could help breeding specific traits in modern watermelons.

## Materials and Methods

### Sampling Design

We sampled individuals from all seven species of *Citrullus*, namely *C. amarus*, *C. colocynthis*, *C. ecirrhosus*, *C. lanatus* (ssp. *cordophanus* and ssp. *vulgaris*), *C. mucosospermus*, *C. naudinianus*, and *C. rehmii*. The 73 resequenced genomes of inbred lines in our data set are mainly from the breeding programs of the Germplasm Bank of National Engineering Research Centre for Vegetables of China and the National Mid-term Gene Bank for Watermelon and Melon of China (Guo et al. 2019; NCBI Sequence Read Archive accession number SRP188834; vouchering information with images and identifications for these samples are in Renner et al. 2021, online data set S3). The vouchers and geographic information for all samples, including the 47 newly sequenced herbarium specimens and the two ancient DNA samples are shown in supplementary table S2, Supplementary Material online.

### Radiocarbon (C-14) Dating of Archaeological Remains

Seeds from site 2-R-65 (Amara West Project, sample AS-814) collected and identified by P. Ryan, seeds from Uan Muhuggiag (UMB-6) collected and identified by K. Wasylikowa (Wasylikowa and van der Veen 2004), and a fragment of a leaf collected by G. Schweinfurth (1836–1925) in one of the tombs at Deir el-Bahari near Luxor and currently held in the Economic Botany Collection at the Royal Botanic Gardens, Kew (specimen 40730), where it was labeled as having come from the coffin of “Unknown Man C” (Cairo museum mummy CG61067), were sent to the Laboratory of Ion Beam Physics, ETH Zurich, where their radiocarbon ages were calculated using the measured ^14^C content after correction for standards, blank values, and fractionation (*δ*^13^C values were measured semi-simultaneously on graphite). The reported conventional age in years BP (before 1950 AD or CE) was calibrated to a calendar age using OxCal version 4.3.2 (Bronk Ramsey 2017) and the IntCal13 atmospheric curve (Reimer et al. 2013; supplementary fig. S1, Supplementary Material online).

### DNA Isolation and Sequencing of Ancient and Historical *Citrullus*

DNA extraction for the two ancient seed samples and the presumed ancient leaf was performed following Dabney et al. (2013) and Wales et al. (2014). For the digestion treatment, a lysis buffer containing 0.5% (w/v) *N*-lauroylsarcosine (Sigma-Aldrich L9150-50G), 50 mM Tris-HCl (Thermo Fisher Scientific 15568025), 20 mM EDTA (VWR E177-500MLDB), 150 mM NaCl (Thermo Fisher Scientific AM9760G), 3.3% 2-mercaptoethanol (Sigma-Aldrich 63689-25ML-F), 50 mM dl-dithiothreitol (Sigma-Aldrich D9779-250MG), and 0.25 mg/mL proteinase K (Promega V3021) was applied to the leaflet powder as described in Wales et al. (2014). DNA purification was performed according to Dabney et al. (2013) with reduced centrifugation speed (450 × g).

Library construction and genome sequencing of the ancient DNA extracts were performed in the aDNA facility of the University in Potsdam; negative controls were included in all steps. DNA extracts were converted to an Illumina sequencing library using the single-strand approach described in Korlević et al. (2015). The protocol included the treatment with uracil-DNA-glycolase (New England Biolabs M0279) to remove Uracil residues and with endonuclease VIII (New England Biolabs M0299) to cleave DNA strands at abasic sites. About 2.5 IU/µL of circligase II (Biozym 131406) was used for the fill-in reaction and carried out overnight. A quantitative polymerase chain reaction (PCR) was performed on a PikoReal 96 Real-Time PCR machine (Thermo Fisher Scientific TCR0096) using 0.2% of the unamplified library following this thermal profile: 10 min initial denaturation step at 95 °C, followed by 40 cycles of: 15 s at 95 °C, 30 s at 60 °C, and 1 min at 72 °C. The quantitative PCR reaction mix contained a final volume of 10 µL: 1 µL of diluted library, 1× SYBR Green qPCR Master Mix (Applied Biosystems 4309155), 0.5 µM of each primer IS7 and IS8. Three replicates of each library were used. Indexing PCR was performed by the appropriate number of cycles determined by qPCR and adding 8 bp indices to 5′ and 3′ adapters. DNA sequencing was performed on an Illumina NextSeq 500 sequencing platform, using the 500/550 High Output v2 kit (75 cycles, Illumina FC-404-2005), with a custom read-1 (Gansauge and Meyer 2013) and a custom index-2 (Paijmans et al. 2017) sequencing primer.

DNA from the 47 historical *Citrullus* samples was extracted from 1 to 2 cm^2^ of leaf tissue from the herbarium specimens (all from Kew herbarium [K], see supplementary table S2, Supplementary Material online for vouchers). The extractions were performed with Qiagen DNeasy Plant kits in the Jodrell Laboratory, Royal Botanic Gardens, Kew. Sequencing was performed by BGI (Hong Kong) using the BGISEQ-500 platform, using paired-end reads of 100 bp.

### Raw Read Data Processing and Mapping

The Illumina raw reads of the 47 herbarium leaf samples were quality-filtered and trimmed using Cutadapt 1.9.1 (Martin 2011), discarding sequences with averaged phred33 scores below 20. Pre- and post-trimming read quality was assessed using FASTQC v.0.1 (Andrews 2010). We used one nuclear reference genome (accessions 97103 [v2]; Guo et al. 2019) and one plastid reference genome (NC032008.1; Zhu et al. 2016) of *C. lanatus* ssp. *vulgaris.* Filtered reads of modern accessions were then mapped to the reference genomes using *BWA 0.7.15* (Li et al. 2009). Duplicate reads were removed and local realignment was performed using *GATK 3.8.0* (McKenna et al. 2010) and the toolkit *picard 1.92* (http://broadinstitute.github.io/picard/). Mean coverage per 10 Kb windows was calculated using *Mosdepth 0.3.0* (Pedersen and Quinlan 2018). Average coverage per base in all samples included in this study was calculated using the depth function of the samtools software (Li et al. 2008) and is provided in supplementary table S1, Supplementary Material online.

### Ancient DNA Authentication, Raw Read Processing, and Mapping

Filtered reads of the two ancient seeds and the Schweinfurth leaf were mapped and aligned against the reference genomes (see Raw Read Data Processing and Mapping) using the pipeline PALEOMIX v.1.2.13 (Schubert et al. 2014). To assess whether the DNA reads in the C-14-confirmed ancient samples AS-814 (Sudan) and UMB-6 (Libya) had patterns consistent with post-mortem DNA damage characteristic of degraded plant tissue, we estimated the proportion and position of deaminated (mis-incorporated) nucleotides in the DNA of the ancient samples by processing aligned aDNA read data against the nuclear reference genome with the software mapDamage2 v.2.0.9 (Jónsson et al. 2013). Based on the patterns of DNA damage observed in the aDNA reads near the first positions of both 3′ and 5′ ends, we trimmed the first three bases of each read prior to mapping. Subsequently, trimmed and quality-filtered aDNA reads were mapped using the software *BWA 0.7.15* (Li et al. 2009) and the *backtrack* algorithm, which is more suitable for reads shorter than 70 bp. Aligned reads were subjected to a realigning step around indels and duplicate filtering as implemented in the software GATK v.3.8.1. (DePristo et al. 2011) and Picard v.1.137 (http://broadinstitute.github.io/picard/) (http://picard.source-forge.net). Following Latorre et al. (2020), the proportion of endogenous DNA content recovered for the ancient seeds was derived from the number of reads mapped against the reference genomes, including PCR duplicates. Number of reads, PCR duplicates, unique hits, and average coverage of ancient DNA accessions are provided in supplementary table S1, Supplementary Material online.

### Ancient DNA Alignment Damage Assessment and Base Quality Recalibration

To account for potential bias that mis-incorporations could introduce in downstream analyses whenever the ancient seed DNA samples were included, we recalibrated base quality scores of individual bases, following the damage patterns estimated earlier for DNA alignments of both the ancient seed accessions and Schweinfurth’s leaf. This was implemented in the software mapDamage2 (option –*rescale*).

If such damage patterns were persistent, the ratio of transitions to transversions in sequence data should be substantially (several-fold) elevated (Stiller et al. 2006). Even a more moderately elevated ratio could be indicative of damage that could skew downstream results. Therefore, we computed the transition to transversion (Ti/Tv) ratio for both ancient seed base-recalibrated alignments. In each case, we constrained the analysis to positions genotyped exclusively in the respective ancient sample. We used seven modern *C. mucosospermus* samples as a reference set to compare the ancient sample Ti/Tiv ratios to. Due to low sequencing depth, we employed a pseudohaplotype approach, using ANGSD, v. 0.9.929 (Korneliussen et al. 2014) to call variant sites to base this analysis on, followed by generating pseudohaploid sequence from which the sites could be extracted. In the first step, variant sites for the seven modern *C. mucosospermus* samples were called if they passed ANGSD filters of: minimum base quality score of 30, minimum mapping quality of 20, minimum depth of 8 and SNP *P*-value of 1 − *e*^3^, and this set of variants was constrained to only those sites representing each respective ancient sample. Missing data in the modern samples comprised up to 12% (AS-814 SNP set) and 2% (UMB6 SNP set). In the second step, we generated consensus pseudohaplotype sequences using -doCounts in ANGSD, followed by random sampling of alleles to generate pseudohaplotype sequence in Consensify v.0.1 (Barlow et al. 2020) and extracted these sites from fasta sequences using bedtools. By calling pseudohaplotype consensus sequence in the same manner as in the phylogenomic analysis (see Nuclear and Plastid Phylogenomics of Citrullus), we conducted a direct validation of the ancient genotypes used in the phylogeny.

To further ensure that the aDNA accessions were not saturated with mis-incorporations, we computed the error rates (i.e., excess of derived alleles) of three recent accessions of *C. mucosospermus* (S17 [collected 1866], S33 [collected in 1908], S37 [collected in 2002]; supplementary fig. S1, Supplementary Material online) and the two aDNA accessions (UMB-6 and AS-814), using the function -doAncError in the ANGSD software and considering all reads for the error estimation (option -1). As input, we employed the alignment files produced by mapping reads against a plastid genome of reference (NC032008.1) as well as a reference sequence of a modern individual of *C. mucosospermus* (sample S37), which was also produced in ANGSD, using the function *doFasta* by sampling randomly a base at each position (option -1) and considering only bases that had a quality score >30 and positions with a sequencing depth of 15×.

### Nuclear and Plastid Phylogenomics of *Citrullus*

To investigate the phylogenetic relationships of populations of *C. lanatus* and closely related taxa, we generated whole-plastid and nuclear-genome-wide DNA matrices that included *C. amarus* (11 individuals), *C. colocynthis* (3 individuals), *C. ecirrhosus* (1 individual), *C. lanatus* ssp. *vulgaris* (78 individuals as well as Schweinfurth’s leaf), *C. lanatus* ssp. *cordophanus* (6 individuals), *C. mucosospermus* (12 individuals from West Africa and 10 from East Africa, viz. S9, S14, S20, S22, S15, S29, WM14, WM21, WM24, WM42), *C. naudinianus* (1 individual), *C. rehmii* (1 individual), one sample from Ethiopia with unclear affinity (WM459), and one of the two ancient DNA samples (UMB-6; the Sudanese seed AS-814 could not be placed in the tree because of too little endogenous DNA). To account for multi-allelic positions in nuclear and plastid genomic regions, the alignments were produced by independently generating pseudo-haploidized consensus sequences of the entire plastid genome (156,906 bp) and 11 nuclear pseudo-molecules representing 363 Mb (∼85% of the total genome size) for each individual. The pseudo-haploidized sequences of modern accessions were generated in ANGSD, using the function *doFasta* and the same quality score settings as above.

The plastid genome alignment was analyzed in an ML framework, using the software RAxML v.8.0 (Stamatakis 2014) with 500 bootstrap replicates, a general time reversible substitution model and 25 gamma rate categories per site. The nuclear pseudo-molecule alignments were analyzed with a combination of ML and MultiSpecies Coalescent methods. Each pseudo-molecule alignment was first filtered by excluding any position that contained >10% of missing data (options -m *gappy* and -g *0.1*) as implemented in the program ClipKIT v.1.1.3 (Steenwyk et al. 2020). The 11 reduced pseudo-molecule alignments, representing ∼4% of the nuclear genome (14.3 Mb), were then fragmented into 10,000-bp-long non-overlapping alignments, using a customized python script (available at https://gist.github.com/jonchang/34c2e8e473ec2e8f50574671e62c3365). An ML phylogeny was produced from each 10 Kb block, using the same settings as used above for the plastid genome ML tree, which resulted in 1,431 gene trees that were summarized into an MSC phylogeny in the software ASTRAL-III v5.6 (Zhang et al. 2018) after collapsing branches with likelihood bootstrap support values <10 as implemented in the function *nw_ed* of the program newick utils toolkit (https://github.com/tjunier/newick_utils). In both nuclear and plastid phylogenomic analyses, trees were rooted on *C. naudinianus* based on Chomicki and Renner (2015). The proportions of informative sites, missing data, and number of positions in nuclear and plastid alignments are provided in supplementary table S4, Supplementary Material online.

To quantify discordance in the nuclear genome, we characterized the tree space of the 1,431 ML gene trees and the 11 MSC trees derived from each pseudo-molecule alignment by first comparing the normalized Quartet (*Q*) scores produced by ASTRAL-III for each pseudo-molecule MSC tree. Subsequently, we computed the topological distances of each rooted ML and pseudo-molecule MSC tree using the Kendall-Colijn metric vector (Kendall and Colijn 2016) and the resulting distance matrix was then subjected to a PCA analysis, as implemented in the R-package TreeSpace v.1.4.1 (https://github.com/thibautjombart/treespace).

### Population Structure, Relatedness, and Gene Flow in *Citrullus*

To trace the genetic ancestry and relatedness of modern and ancient samples of *Citrullus*, we relied on the computation of GLs from reads mapped against the nuclear and plastid reference genome as implemented in the ANGSD software (above). We estimated minor and major allele frequencies (option -doMajorMinor 1) using the GATK GL model (option -GL *2*), a base quality of 30 (option -minQ *30*), and a polymorphic site filtering value of *P* = 1 − *e*^6^ (-SNP_pval *1e-6*), a default maf filtering of 0.05, while trimming the first three bases at the 5′ and 3′ ends of each read to account for the presence of remaining mis-incorporations (option -trim *3*) for all 47 herbarium specimens. To account for the potential bias introduced by missing data in downstream analyses dependent on GLs, we also estimated GLs using the same parameters as above but keeping sites that were present in all individuals (option -minInd *131*).

The estimated GLs were used to conduct PCAs, infer nuclear genomic admixture, and test for introgression. PCA relied on the software PCansgd v.0.982 (Meisner and Albrechtsen 2018) by computing a covariance matrix from the plastid and nuclear GL input files and setting a maximum of 1,000 iterations. Admixture analyses were performed using the software NGSadmix v32 by clustering individuals into *K* ancestral groups with *K* from 2 to 8, using variant sites with a minor allele frequency >0.05. Markov Chain Monte Carlo analyses were run for each analysis using up to 100,000 iterations and until the log likelihood difference in 50 iterations was below 0.05, and with a tolerance of convergence of 1 × 10^−5^.

To test for introgression, we used Patterson’s D-statistics, also known as the ABBA-BABA test (Kulathinal et al. 2009; Green et al. 2010; Durand et al. 2011). This test (conducted in ANGSD v.0.9.929) is based on the counts of the number of bi-allelic sites that have a topology different from the species tree, which is assumed as known. The null hypothesis of incomplete lineage sorting driving discordance is accepted when the proportion of ABBA and BABA sites is similar (*Z* score = 0). Under gene flow, a bias in the proportion of ABBA and BABA sites is expected (positive or negative *Z* score). The analysis was conducted by randomly sampling one base per position (option -doAbbababa *1*), removing all transitions to account for potential remaining deaminated bases in the aDNA samples (option -rmTrans *1*) and considering only sites that had a minimum and a maximum sequencing depth of 1 (-setMinDepth 1) and a 100 (-setMaxDepth 100), respectively. Additionally, to further account for potential biases on artificial gene flow patterns between the aDNA samples and modern specimens driven by DNA damage whenever the aDNA samples were evaluated on *H1* position (Barlow et al. 2020), we also conducted the ABBA-BABA test using the same settings as specified above, but considering only non-polymorphic sites (option *-enhance* 1). Significant evidence of admixture was tested using a weighted block jackknife with 1 Mb non-overlapping blocks (Efron 1981). We considered *Z* scores higher than 3 or smaller than −3 to be significant. The populations considered for this analysis were *C. amarus* (10 specimens), *C. lanatus* ssp. *vulgaris* (27 specimens), *C. lanatus* ssp. *cordophanus* (4 specimens), *C. mucosospermus* (6 specimens from West Africa, 4 from East Africa, viz. S14, S20, S22, S15, S29), and in some runs also the two aDNA accessions. With these groups, we tested for (1) admixture between *C. mucosospermus* and *C. lanatus* ssp. *cordophanus*, (2) admixture between *C. mucosospermus* and *C. lanatus* ssp. *vulgaris*, (3) admixture between *C. amarus* and *C. lanatus* ssp. *vulgaris*, and (4) admixture between the aDNA accessions and *C. lanatus* ssp. *cordophanus*, *C. lanatus* ssp. *vulgaris*, and *C. amarus*.

## Supplementary Material

msac168_Supplementary_Data

## Data Availability

All sequences are available at BioProject PRJNA856800 and PRJNA857275.
