# Supplementary material for: Genome Sequencing of up to 6,000-Year-Old Citrullus Seeds Reveals Use of a Bitter-Fleshed Species Prior to Watermelon Domestication
Source: Mol Biol Evol. 2022 Jul 30;39(8):msac168. doi: 10.1093/molbev/msac168 (PMC9387916; doi:10.1093/molbev/msac168)
Supplement: msac168_Supplementary_Data [file msac168_supplementary_data.zip › Pérez-Escobar_et_al_Citrullus_MBE_FigsS1-S6.pdf]

Molecular Biology and Evolution - **Supporting Information**

Genome sequencing of up to 6,000-yr-old Citrullus seeds reveals use of a bitter-fleshed species prior to watermelon domestication

Oscar A. Pérez-Escobar,[1†] Sergio Tusso,[2†] Natalia A. S. Przelomska[1], Shan Wu,[3] Philippa Ryan,[1] Mark Nesbitt,[1] Martina V. Silber,[4] Michaela Preick,[5] Zhangjun Fei,[3,6] Michael Hofreiter,[5] Guillaume Chomicki,[7*] and Susanne S. Renner[4,8*]

[1]Royal Botanic Gardens, Kew, TW9 3AE, United Kingdom
[2]Faculty of Biology, Division of Genetics, University of Munich (LMU), 82152 Planegg- Martinsried, Germany
[3]Boyce Thompson Institute, Ithaca, NY 14853, USA
[4]Faculty of Biology, Systematic Botany and Mycology, University of Munich (LMU), 80638 Munich, Germany
[5]Faculty of Mathematics and Natural Sciences, Institute for Biochemistry and Biology, University of Potsdam, 14476 Potsdam, Germany
[6]USDA-ARS, Robert W. Holley Center for Agriculture and Health, Ithaca, NY 14853, USA
[7]Ecology and Evolutionary Biology, School of Bioscience, University of Sheffield, Western Bank, Sheffield, S10 2TN, United Kingdom
[8]Department of Biology, Washington University, Saint Louis, MO 63130, USA

†These authors contributed equally to this work.

[*]Shared senior authorship and authors for correspondence: E-mail: srenner@wustl.edu, g.chomicki@sheffield.ac.uk

The following Supporting Information is available for this article:

Supplementary Figures S1-S6
Supplementary Tables S1-S4

Supplementary Figures

Figure S1. C-14 dating. (A) Libyan seed from Uan Muhuggiag (UMB-6), first dated in Wolcott et al. (2021). (B) Sudanese seed from Amara West (AS-814). (C) Schweinfurth's leaf (collection at the Royal Botanic Gardens, Kew, specimen 40730).

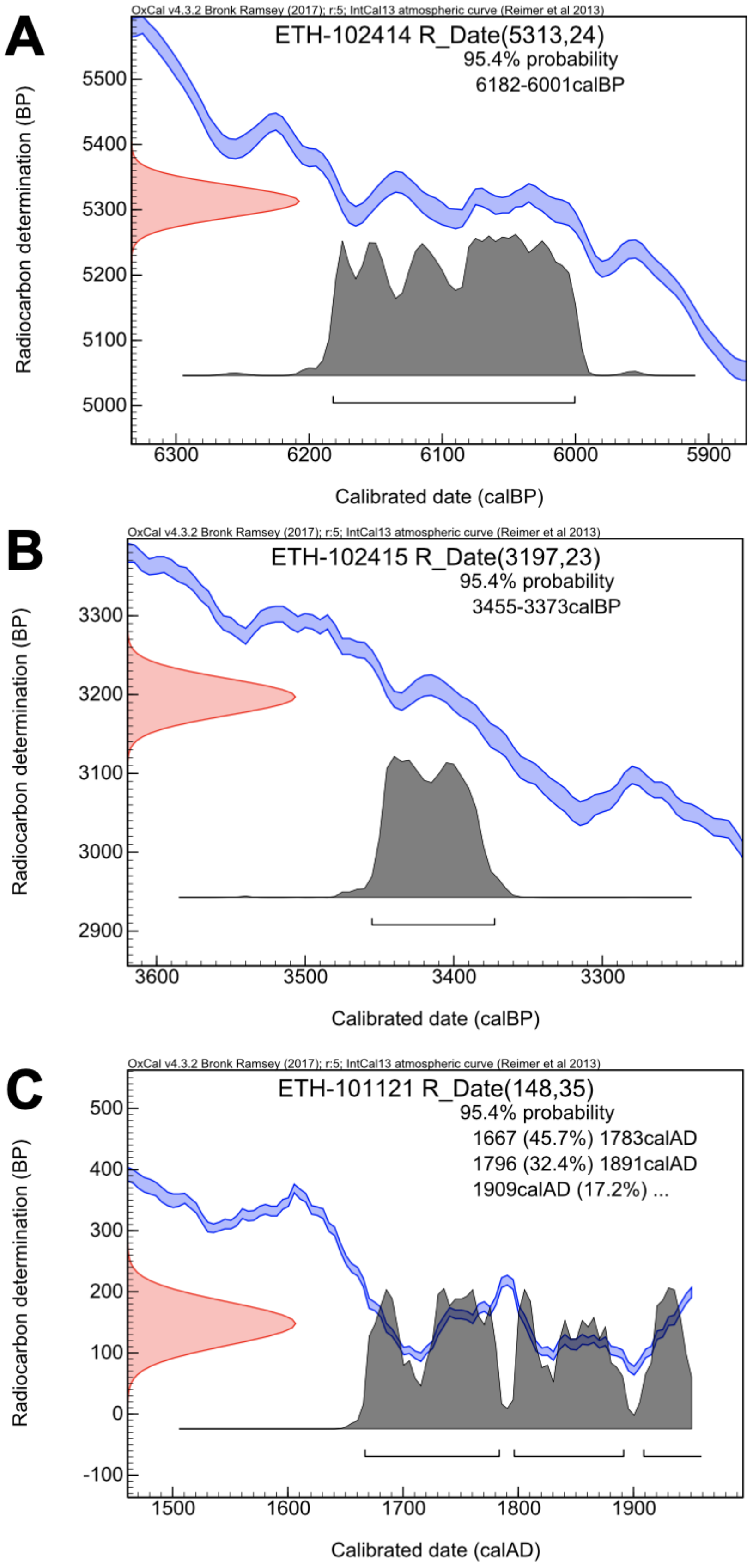

Figure S2. Comparison of error rates in the ancient DNA samples with more recent *Citrullus mucosospermus* accessions (S17 [collected 1866], S33 [collected in 1908], S37 [collected in 2002]). (A) Comparisons of substitution rates. This analysis shows that C>T and G>A misincorporations are not disproportionally elevated in the ancient samples. (B) Overall error rates (i.e, excess of derived alleles), computed after read trimming and quality base recalibration. (C-D) Authentication of the ancient DNA samples (AS-814 and UMB-6, respectively), showing DNA misincorporations in function of position in the reads, the distribution of read length, and the cumulative frequencies of C->T and G->A substitutions.

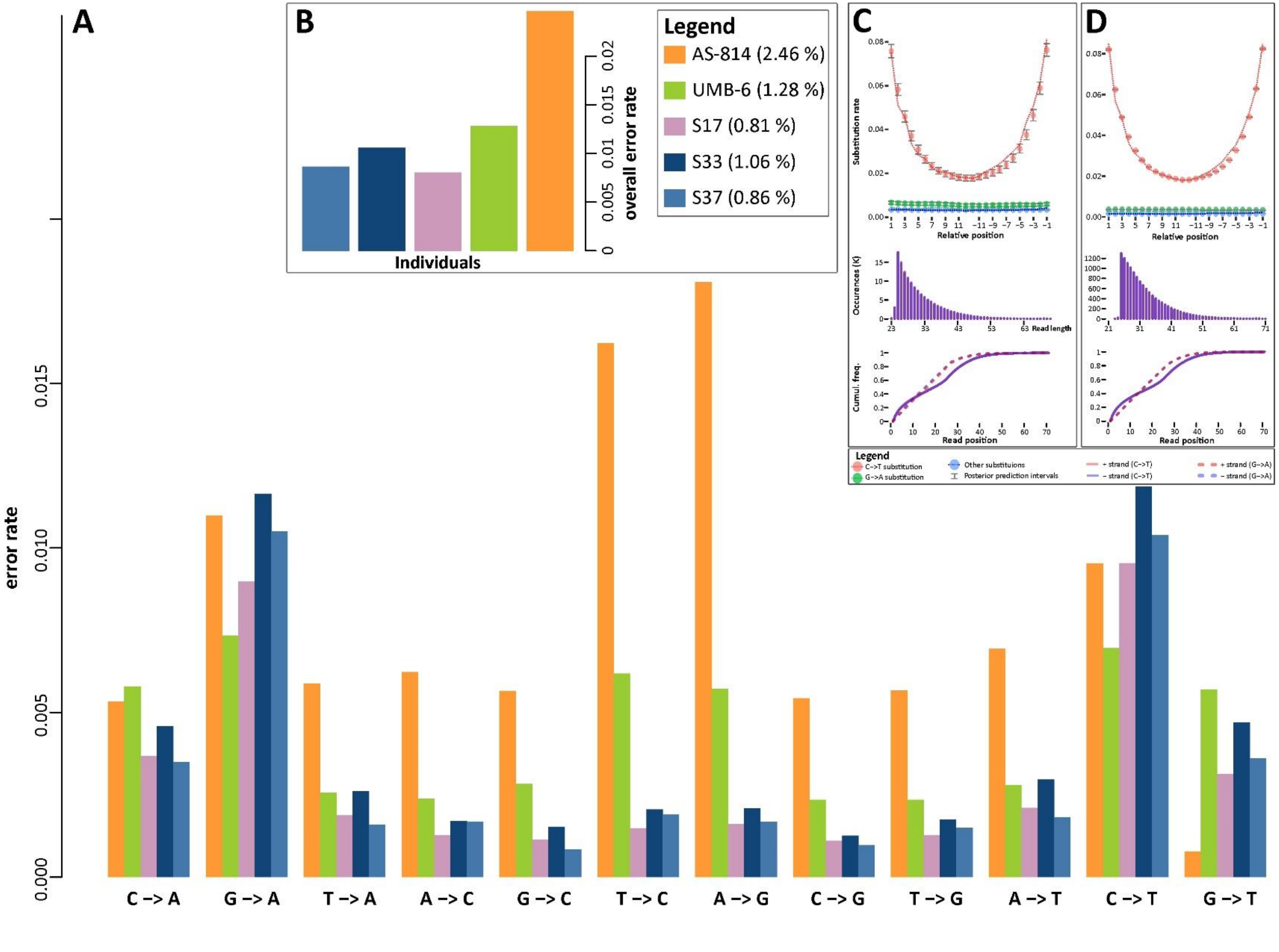

Figure S3. Tanglegram showing phylogenies from the nuclear (left) biparentally inherited genome and the plastid maternal genome (right), showing gene flow patterns across *Citrullus*. Only the most statistically supported incongruences are shown.

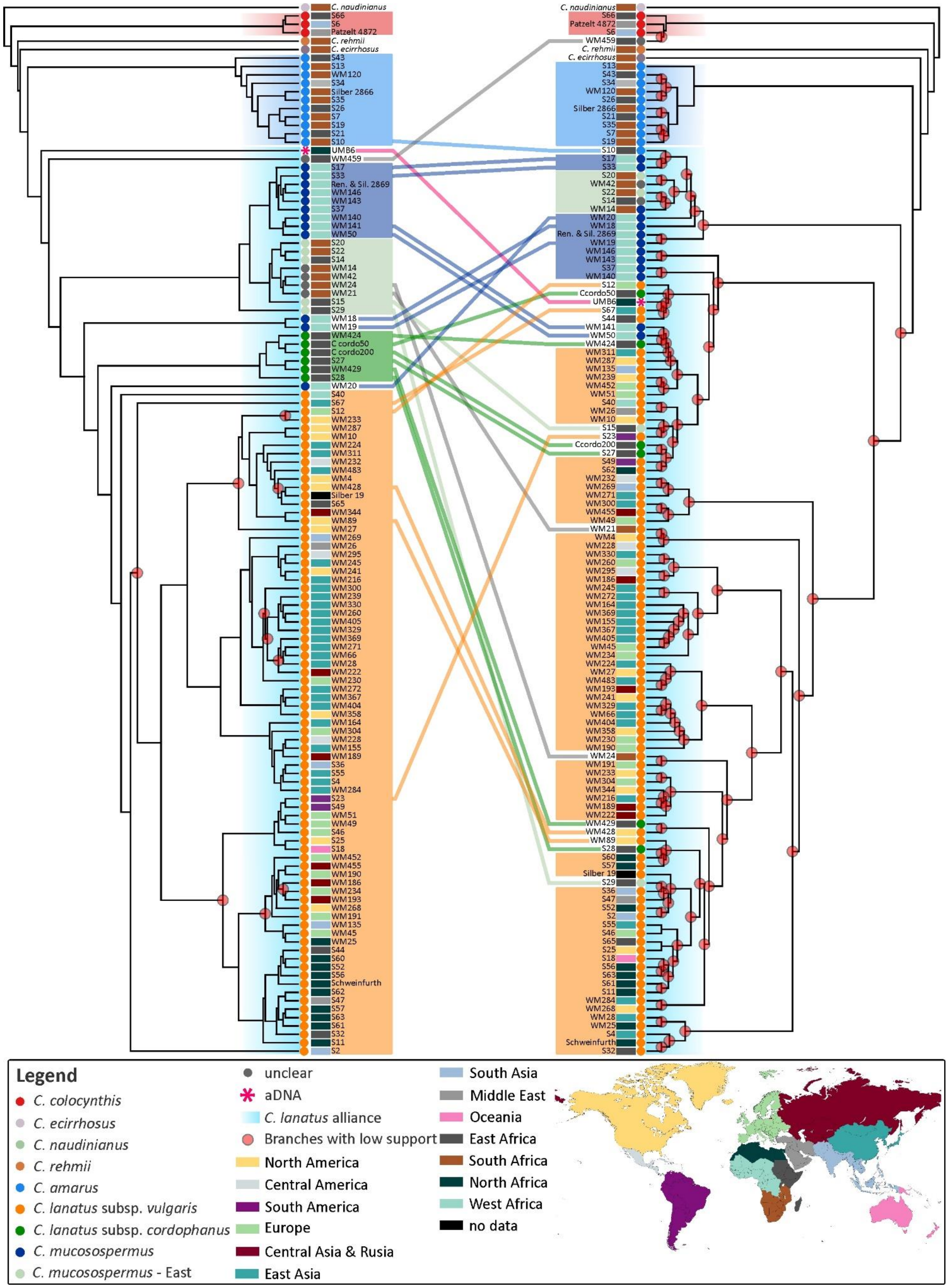

Figure S4. PCA of the nuclear genotype likelihoods (GL) produced from a non-missing GLs dataset (A) and (B) PCA inferred from GLs derived from reads mapped against a plastid reference genome of *C. lanatus* subsp. *vulgaris*.

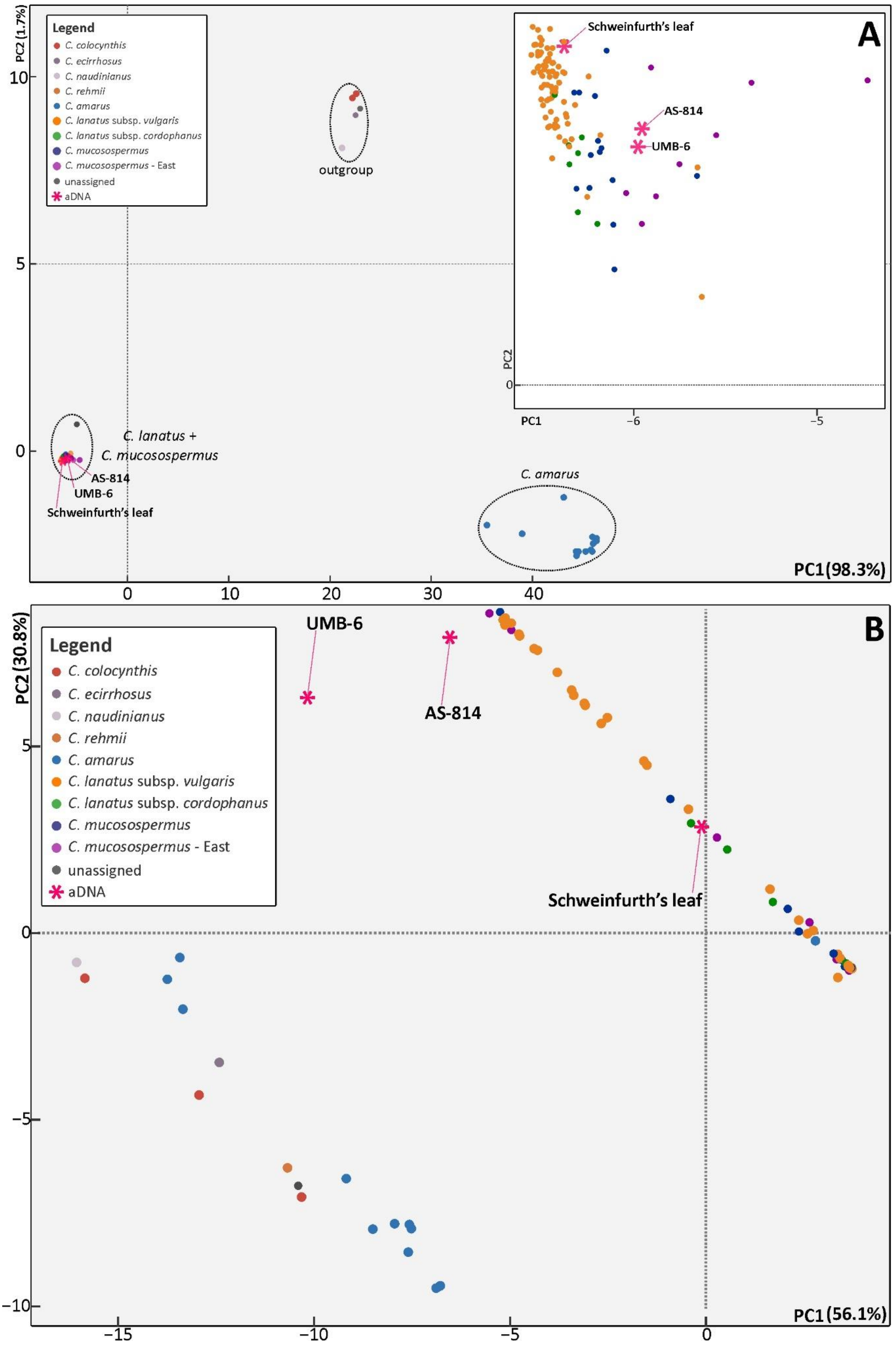

Figure S5. ADMIXTURE plot analyses and their corresponding log – and delta-likelihood inferred from (A) GLs containing missing data (B) and without missing data.

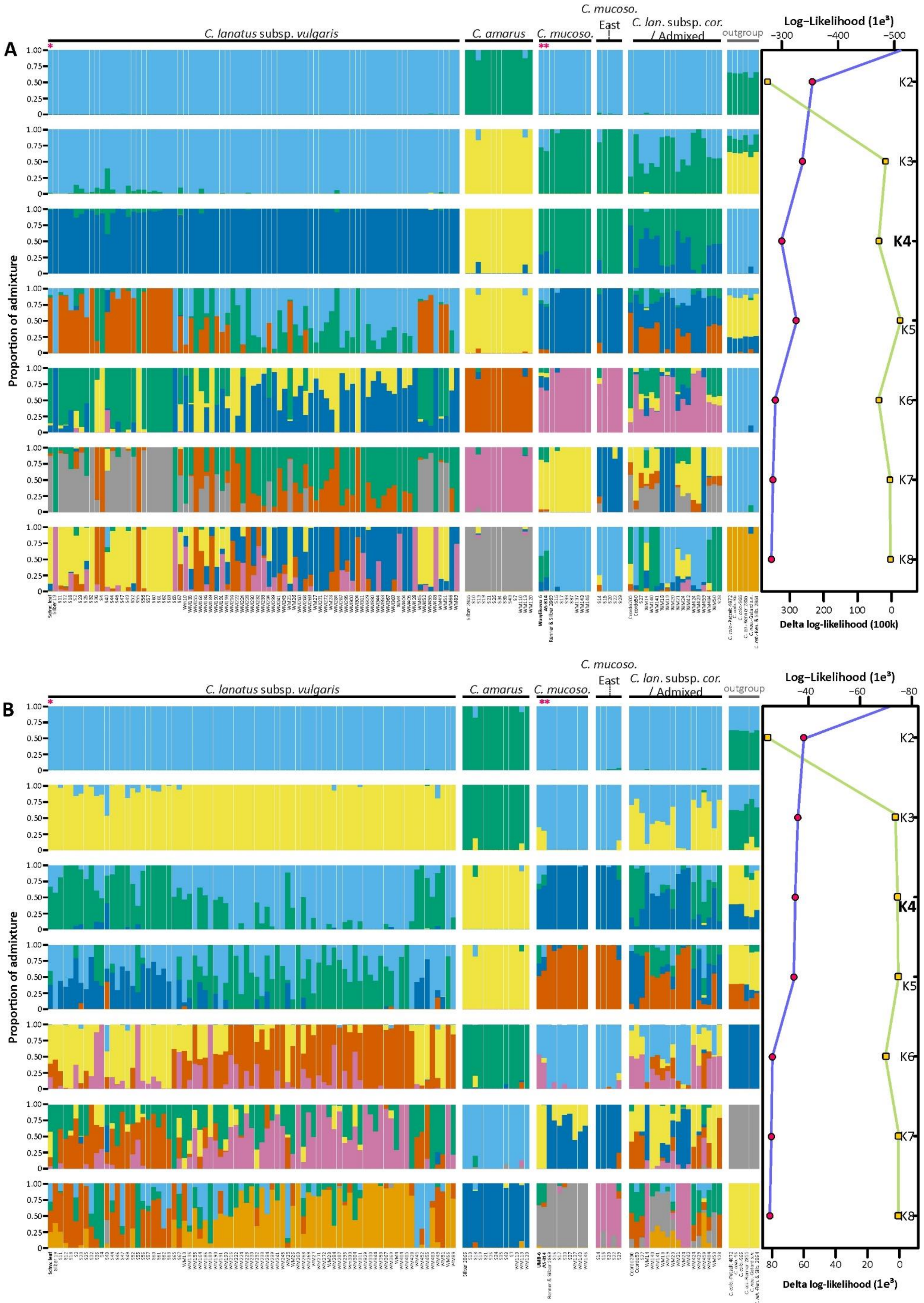

Figure S6. Evolution of fruit bitterness and pulp color in *Citrullus*. (A) Cucurbitacin pathway in *Citrullus* highlighting key changes in functionality of the fruit-specific transcription factor ClBt that controls pulp bitterness. (B) Lycopene pathway in *Citrullus*, highlighting a key substitution in the LYCB (lycopene β-cyclase) gene that is associated with lycopene accumulation controlling fruit color. Abbreviations: PSY: phytoene synthase, PDS: phytoene desaturase, ZDS: zetacarotene desaturase, CRTISO: prolycopene isomerase, LYCB (β-LYC): lycopene β-cyclase. (C) Fruit bitterness and flesh color identified using the key SNPs shown in A and B, mapped across the *Citrullus* phylogeny.

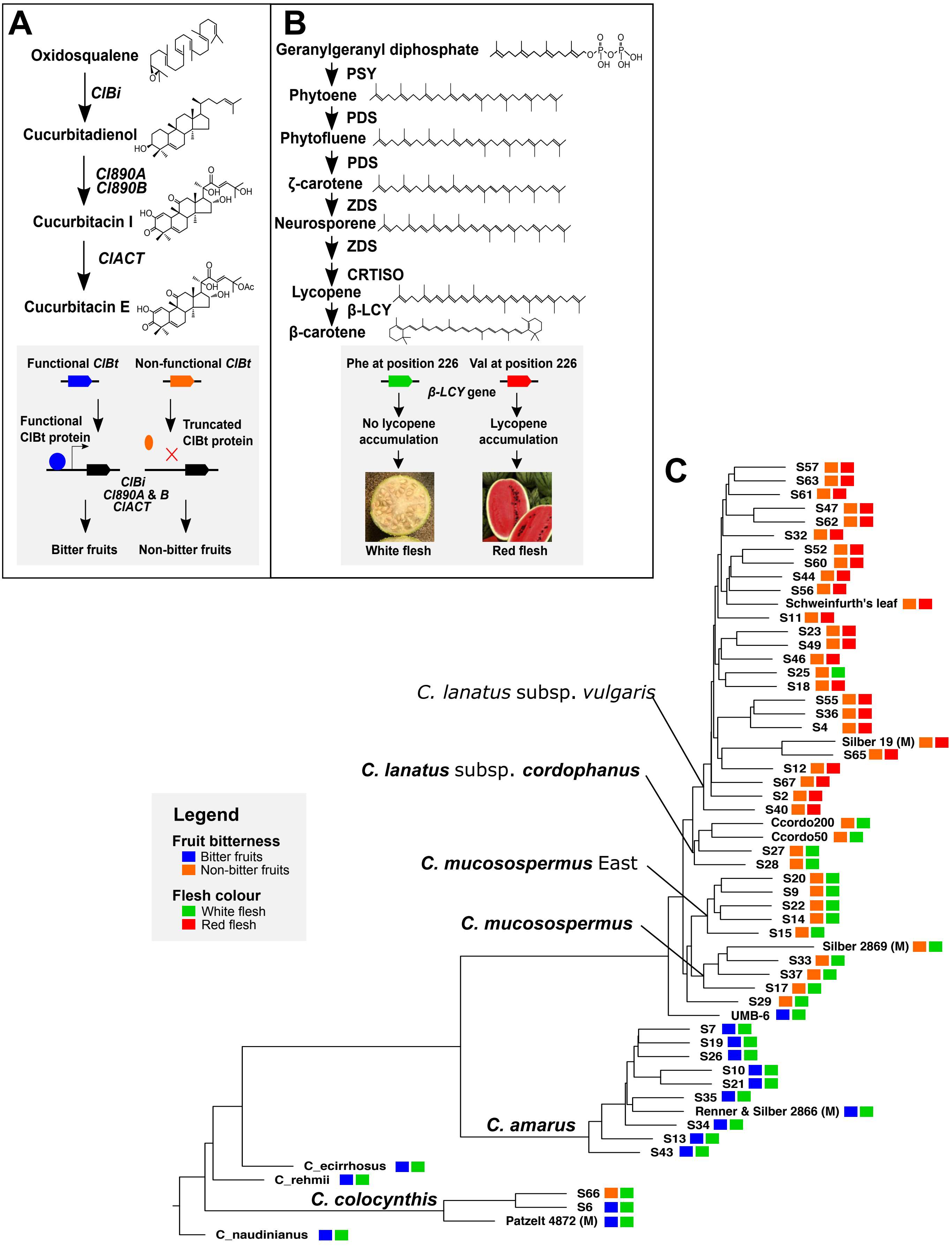

Supplementary tables

Table S1. Sequencing statistics of the samples used in this study. The ancient DNA samples are shown at the end of the table. Due to the size of the table, it has made available at https://doi.org/10.6084/m9.figshare.19620636

Table S2. Specimens used in this study. More ample information on the germplasm specimens is given Supplementary information Dataset S03 in Renner et al. (2021) [https://www.pnas.org/doi/10.1073/pnas.2101486118#supplementary-materials]. Due to the size of the table, it has made available at https://doi.org/10.6084/m9.figshare.19620636

Table S3. ABBA-BABA test considering polymorphic and no- polymorphic sites, and non-polymorphic sites only. Results derived from polymorphic and non-polymorphic sites are highlighted in green, non-polymorphic sites are highlighted on blue. Permutations involving aDNA accessions are highlighted on yellow pale. Clanatus = *C. lanatus* subsp. *vulgaris*; Ccordo = *C. lanatus* subsp. *cordophanus.* Due to the size of the table, it has made available at https://doi.org/10.6084/m9.figshare.19620636

Table S4. Proportion of informative sites, missing data and number of positions in nuclear and plastid alignments. Due to the size of the table, it has made available at https://doi.org/10.6084/m9.figshare.19620636
